# Contemporary Digital Life: Cyberpsychological Perspectives

**DOI:** 10.5964/ejop.v14i4.1795

**Published:** 2018-11-30

**Authors:** Camelia Gradinaru

**Affiliations:** a Department of Interdisciplinary Research in Social Sciences and Humanities, “Alexandru Ioan Cuza” University of Iași, Iași, Romania

[Other p1]The very recent book published by Macmillan is a provocative immersion in contemporary digital experiences and also a meta-theoretical approach of Cyberpsychology, in terms of powers and limits. The three authors of this book are teaching Cyberpsychology at the University of Brighton, their competencies being easily identifiable in the structure of the book. Dave Harley has as main research topics Cyberpsychology and Human Computer Interaction, with a particular interest in older people’s use of digital media. Julie Morgan focuses on autobiographical memory, social anxiety and the development of social fears, and the social dimension of nature-connectedness within mental health recovery. Hannah Frith centres her research on sexuality, embodiment and subjectivity.

This review will provide an overview of the volume, a list of shifts and trend lines advanced by the authors, and also some questions raised by the book.

First, the authors felicitously put together a *structure* that is more inclusive than other papers dedicated to Cyberpsychology. Instead of focusing on a certain subject or age category, the topics are chronologically developed, from childhood to older age, emphasising key aspects for every stage. The role of new technologies is longitudinally studied, searching for the right place that new media have in this complex puzzle of our lives. Thus, the relationship with technology is constantly changing, emerging from different needs, and creating diverse subjective and contextual meanings. The book explores the experience of “growing up online” for children and adolescents (Chapter 2), aspects of online activities that transcend age – such as online identity, online social relationships, cybersex, trolls, and being alone online (Chapters 3-7) – and also the experiences of older people engaged in digital processes (Chapter 8). One recent research area is represented by the modalities in which dying and grieving are mediated online (Chapter 9). As Larissa Hjorth and Kathleen M. Cumiskey discussed in their last contribution to *A Networked Self and Platforms, Stories, Connections,* edited by Zizi Papacharissi (2018), the images of dead, especially the selfies at funeral, became an affective witnessing media. Online practices of memorialisation could change the social rules about grieving, or could introduce new rites. The first and the last chapter are reflections about the subjective kinds of experiencing online contexts and about the embedded digital way in which we live today.

Second, I truly appreciated the strong standpoint of the authors, who opt for an approach that tries to surpass the general “disease” of *polarization*. A lot of research on human – technology interactions is extremist, devoid of nuances, and applicable to a small number of situations. The disjunctive logic of “or – or” is given up in favour of an inclusive logic of “and – and”. The phenomena are too complex to be interpreted as easy to categorize or to generalise. In this vein, Harley, Morgan, and Frith always look up for both bad and good sides of using technology in a particular context, emphasising the common misinterpretations encountered in literature review. The historical perspective about media and technology is a good treatment for the predisposition to good – bad judgements. Every new medium was initially perceived as an imminent danger for the human sanity, physical wellbeing or social presence. In my view, Patrice Flichy’s concept of “technological imaginary” (Flichy, 2003) represents a useful lens through which we can explain this reaction in front of a novelty. Also, the Greek word “pharmakon” includes the quintessence of the concerns related to the effects of new technology on the human beings, society and culture. Thus, the technology is conceived both as a remedy and a poison, a positive and a negative thing, a perfect scapegoat for many types of decline. Multiple inventions are ambivalent in terms of their uses ‒ they can help and destruct in the same time. Instead of negative or positive approaches, pharmacological interpretations are more comprehensive and “realistic”.

Third, the authors clearly appreciate the role of *context* for an adequate understanding of the various modalities in which people use digital technologies. The situated, contextual approaches are antidotes for the simplistic way to judge the multifarious experiences that people have within new media settings. Even if the “context collapse” (Marwick & Boyd, 2011) is a known reality of new media – social media melts diverse contexts into one, bringing together different audiences, and making difficult the negotiation of interactions – Harley, Morgan, and Frith underline the need for more subjective and nuanced approaches. The way in which they conceive context is more general, including not only social media but all the digital experiences that a user can encounter at the interface with technology. They privileged an edifying path of integrating the most important factors such as age, life orientation, level of digital literacy, socio-cultural data. Thus, the authors are strongly advocating the value of contextual perspectives in attempting to make sense of our digital lives, by rejecting the general biases, presuppositions and oversimplifications in this domain. Thus, the deterministic views are challenged in favour of an alternative perspective that values a more subjective and situated dynamic of relationships between humans and technology and a more phenomenological and ethnographic methodology.

Fourth, the authors re-problematize the concept of *causality*, its misunderstandings and fallacious uses. Cyberpsychology research has been following for too much time the main principle that new technologies cause a plethora of bad effects on our psyche, so that the testing of this hypothesis seems to be mandatory. As a logical consequence, the causality itself and its use in Cyberpsychology are to be verified. The “media effects” theory in line with positivist views on Cyberpsychology constructed a strong “technologically deterministic agenda” that exaggerated the role of technology, transforming in the same time the user in a passive, vulnerable, and overstimulated recipient. In spite of being a philosophical consecrated term, the concept of causality underlies many of these assertions, so its validity has to be checked every time, in every area. Moreover, Harley, Morgan, and Frith prefer a cyberpsychological view that moves research “beyond the blaming of technology for social and psychological ‘effects’ and towards a greater awareness of social context and subjectivity as significant in explaining digital technology use” (p. 241).

Fifth, a central concern is the *practices of meaning-making* in the context of digital technology. Proving once again that Cyberpsychology represents an interdisciplinary realm of study, the researchers insist on the great value of meaning for our lives and on the ways in which digital technology contributes to the emerging of personal, social, political, cultural meanings in the contemporary techno-sphere. Individuals engage differently with new media and make sense personally to the mediated experiences and interactions. We all search for meaning and assigning meaning to our lives constitutes a main quest for us. The question is whether the Internet and the online medium could provide some significant ways of meaning-making for people living in the 21^st^ century. Instead of searching for trends that can be generalized over populations, the user experience research could better capture the motivations that informs the everyday use of technology. Thus, life stages are essential in defining the meanings that arise in symbiosis with technology, from childhood to the old age, because every stage has some dominant characteristics. Moreover, in order to grasp the practices of meaning-making we have to renounce the dichotomy between “real life” and online life. The pervasiveness of mobile communication transformed our everyday experiences and our references to digital platforms in such a manner that become hard to separate the offline from the online. Of course, this makes things difficult for the researcher that has the complicated task to reunite data derived from both “spaces”.

Sixth, what are the *theoretical implications* of the assumptions advanced in this book for Cyberpsychology as a discipline? What is the meta-theoretical discourse sustained by Harley, Morgan, and Frith? I have to point out the authors’ desire for radical remodelling, the entire volume being a set of arguments that plead together for alternative approaches. The aim of the book is a change of paradigm from quantitative, experimental, deterministic perspectives towards qualitative, subjective, ethnographic views. Also, the interdisciplinary collaboration is discussed, cyberpsychological research has to incorporate studies from other disciplines such as anthropology, sociology, media studies, human-computer interaction and other related fields. Cyberpsychology studies should not be restricted only to the identification of the potential risks or negative traits but also they should explain the underlying processes. They have to do more than detect possible trends or causes for different behaviours, they have to be apt to capture how Internet-based platforms become meaningful for such diverse groups. “The subjective dimensions of meaning” (p. 230) seems to be a better route for Cyberpsychology in the view of the authors, because it provides an understanding “from the inside”. Thus, the experiences of people alongside the deep comprehension of context direct the readers to a more nuanced and authentic look at the interplay between individuals and the digital realm.

Seventh, the question that we have to raise is whether such a seductive and reforming perspective could hold. The authors did not show how this alternate approach could be translated in efficient methodological ways of research. They indicate valuable paths for Cyberpsychology but they did not really test their conjectures. Theoretically, their critiques are reasonable, but the complexity of the phenomena seems to overpass the actual methodological powers of Cyberpsychology. The enlargement of the theoretical horizon of Cyberpsychology is desirable, especially in deepening the explanations and the correlations among elements that until now were considered separated. The single concern is if this theoretical perspective could be accurately put into practice.

In conclusion, *Cyberpsychology as Everyday Digital Experience across the Lifespan* is a solid and compelling work that makes itself conspicuous through criticism, inclusiveness, interdisciplinary perspectives, and sharp points of view. This book offers a distinguishable approach to Cyberpsychology, and it will be of great interest to anyone who wants to decipher the complex relationship between technology and our lives.
